# Multistage allocation problem for Mexican pension funds

**DOI:** 10.1371/journal.pone.0249857

**Published:** 2021-04-13

**Authors:** Andrés García-Medina, Norberto A. Hernández-Leandro, Graciela González Farías, Nelson Muriel

**Affiliations:** 1 Consejo Nacional de Ciencia y Tecnología, Ciudad de México, México; 2 Unidad Monterrey, Centro de Investigación en Matemáticas, Apodaca, Nuevo León, México; 3 Probability and Statistics, Centro de Investigación en Matemáticas, Guanajuato, Mexico; 4 Departament of Physics and Mathematics, Universidad Iberoamericana CDMX, Ciudad de México, México; University of Almeria, SPAIN

## Abstract

The problem of multistage allocation is solved using the Target Date Fund (TDF) strategy subject to a set of restrictions which model the latest regulatory framework of the Mexican pension system. The investment trajectory or glide-path for a representative set of 14 assets of heterogeneous characteristics is studied during a 161 quarters long horizon. The expected returns are estimated by the GARCH(1,1), EGARCH(1,1), GJR-GARCH(1,1) models, and a stationary block bootstrap model is used as a benchmark for comparison. A fixed historical covariance matrix and a multi-period estimation of DCC-GARCH(1,1) are also considered as inputs of the objective function. Forecasts are evaluated through their asymmetric dependencies as quantified by the transfer entropy measure. In general, we find very similar glide-paths so that the overall structure of the investment is maintained and does not rely on the particular forecasting model. However, the GARCH(1,1) under a fixed historical covariance matrix exhibits the highest Sharpe ratio and in this sense represents the best trade-off between wealth and risk. As expected, the initial stages of the obtained glide-paths are initially dominated by risky assets and gradually transition into bonds towards the end oof the trajectory. Overall, the methodology proposed here is computationally efficient and displays the desired properties of a TDF strategy in realistic settings.

## 1 Introduction

At present, population aging poses substantial economic challenges to nearly all of the governments of the world. Pension systems face a series of difficulties mainly due to incomplete contribution records, increased life expectancy, and uncertainty over future market fluctuations. Moreover, pension systems must be sustainable, adequate, and equitable to satisfy the demands and preferences of people and offer them a plan suitable for the present era of rapid change. As a consequence, it is necessary to search for a general solution to pension system issues. The main paths towards such a solution proposed thus far are to reduce monthly pensions, to increase voluntary contributions, to delay the retirement age, and to increase worker output [1].

One way in which the pension system has met the challenges mentioned above has been to shift from models in which employees rely on social security under defined benefit (DB) pension plans to a system in which employees must rely on their own savings and investment decisions to fund their retirement, a system known as a defined contribution (DC) plan. In the latter system, employees are responsible for financing their own retirement. Moreover, the current approach to the investment problem is based on life-cycle plans [2, 3], whereby investors should allocate a larger share of their long-term savings to stocks when they are young and decrease this allocation as they approach retirement age. Life-cycle funds reduce the stock exposure of the fund as the target maturity date approaches. Adopting them as default investment options in DC pension plans might help a significant number of individuals, particularly those with lower levels of education, wealth, and income because, as argued in [4], members of these groups exhibit a high degree of inertia in their contribution and investing decisions.

In particular, and to overcome the problems derived from the increase in the elderly population and overall life expectancy, the Mexican pension system has experienced several reforms in recent decades. In 1997, Mexico reformed its DB plan and implemented a DC plan based on a mutual fund system in which the so called Investment Societies Specialized in Retirement Funds (SIEFOREs, for its acronym in Spanish) manage different investment funds with different risk exposure profiles. Until 2004, the official investment policy of the SIEFOREs was restricted to investing only in Mexican government and corporate debt securities; but in that year a new regulation enabled SIEFOREs to include Mexican equity. In 2007, the investment policy changed again and began to follow a life-cycle investment profile. Since then, SIEFOREs have been allowed to invest in international stocks and in real estate. Five categories of SIEFOREs were available until 2019, with type 5 being the riskiest one with a portfolio consisting primarily of equities and international debt, and type 1 being more conservative and diminated by bonds [5]. For a review and analysis of this pension system with data up to 2014, the reader is referred to [6].

At the end of 2019, derived from the new provisions of the regulation of the Retirement Savings System (SAR, for its acronym in Spanish) in Mexico [7], the Retirement Fund Administrators (Afore, for its accronim in Spanish; see [8] for a review of the market of Afores in Mexico) ceased to operate under the multi-fund scheme and was shifted to a model called Target Date Funds (TDF). The main difference between the multi-fund model and TDF is the investment process. In the multi-fund model, at a certain age, the worker migrates between funds, which are characterized by having a different investment regime and a different risk exposure. In TDF, the individual makes an initial investment and continues to contribute throughout his or her working life. The investor’s objective is then to maximize his or her pension benefits during retirement [9]. In TDF, the investment portfolio is restructured reducing its share of risky assets as the worker approaches the retirement date. This implies that the worker’s resources stay in the same fund (unlike the DC system in which funds were transferred from one SIEFORE to another), the long-term investment strategy of which is adjusted with respect to risk exposure and the age of the employee.

A long-term investment strategy, investment trajectory or glide-path, determines the pace at which TDF changes its composition, depending on the risk-return ratio of its constituent assets. In addition to risk, the worker’s retirement date and income prospects at retirement –the replacement rate (RT)– and contributions over time are crucial in determining the allocation trajectory of the fund. Thus, the most recent regulation in Mexico, implemented on the 13th of December 2019, poses a rather interesting dynamic optimization problem whose optimal glide-path is fundamental. Some restrictions are imposed on this glide-path by regulation. For instance, the newly managed funds need to contemplate the savings of the particular generation. Additionally, the glide-path must have a horizon of 161 quarters, which amounts to approximately 40 years. Finally, regulation establishes the investment limits for each asset class in time. For example, a maximum of 60% of the total resources available can be invested in equity during the first quarter; but this figure falls to 15% in the last quarter. The traceability of investments is not defined and depends on various variables inherent to the profile of the workers such as savings rate, contributions, and demographic profile, among others factors.

The new regulation demands a solution that supports the notion that the retirement horizon matters for asset allocation. Thus, we should prefer the approach of a life-cycle pension plan with age-based and risk-based characteristics. Unfortunately, the traditional approach of the mean-variance portfolio analysis of Markowitz [10] does not provide scientific support for the horizon-based investing approach to asset allocation that characterizes life-cycle funds [4]. The reason is that mean-variance analysis assumes that investors live in a parsimonious world of constant risk and return. A TDF pension fund must address the challenges of implementing state-of-the-art life-cycle optimization techniques to incorporate the long-term investment horizon while also accounting for the periodic contributions of the investor to adequately model the pension funds in a TDF system.

In this paper, we attempt to solve the TDF problem for the case of Mexico. To this end, we implement a function that obtains an optimal asset allocation strategy and simultaneously allows maximizing a generation’s target wealth under the investment limits established by the new regulation. In doing so, we choose realistic scenarios considering a set of assets and investment limits that are used in practice and obey the requirements of the new regulation. The general considerations of our analysis are based on the premises of the homogeneity of human capital characteristics, equal risk tolerance for the participants, a single long-term investment option, and tax irrelevance. Under these premises, a single life-cycle fund per retirement horizon is sufficient [4].

More specifically, we model the asset allocation as a multi-period optimization problem in which the objective is to minimize the risk at the end of the investment trajectory while satisfying a lower bound constraint on the total return of investment. This lower bound is in fact related to the desired replacement rate of the investment fund. An optimal frontier is obtained for a set of replacement rates attained with the lowest investment risk in a range of values. We thus obtain a set of optimal solutions, each with a particular glide-path.

The rest of the paper is structured as follows: Section 2 introduces the multiperiod objective function that is used to solve the allocation problem. In section 3, the volatility models to estimate the input of the objective function are described. Transfer entropy theory is briefly discussed in section 4 to analyse the dependencies among the assets. Next, section 5 describes the dataset and the set of constrains used in the multistage allocation problem. Section 6 discusses the methodology to compute the expected returns and covariance matrices, and shows preliminary results on the behavior of the forecasted values and their asymmetric dependencies. The optimized results are shown and interpreted in section 7. Finally, in the concluding section 8, the main findings are summarized, and future research directions are proposed.

## 2 Multistage model

We are interested in solving the asset allocation problem with restrictions under a multiperiod approach. Thus, the objective function must consider the effect of each period on the reallocation of assets within the portfolio and seek to minimize the total risk of the investment, that is, when considering the 161 quarters of the investment fund horizon established by the new regulation in Mexico.

To achieve this objective, we adopt a discrete approach since the continuous approach [2, 3, 11] is practically infeasible in a computational sense when dealing with a large number of variables, periods, and restrictions, as is the case for the pension fund problem that we aim to solve.

Nevertheless, discrete representation also presents problems that can lead to an implementation where the computation time increases exponentially with the number of the periods considered. The difficulty is that a stochastic programming formulation starts from the fact that the decisions made in each stage or period are decision rules conditioned on past events. This problem restricts the applicability of the multiperiod approach to a limited number of assets, periods and restrictions.

Thus, the specific approach that we propose is mainly based on [12], where the multistage asset allocation problem is reduced to a convex quadratic programming problem with linear constraints that can be solved globally and at relatively low computational cost.

### 2.1 Model definition

Let us consider a set of *p* assets and an investment horizon *T* divided into *t* periods of equal duration Δ*t*. Denote by *x*_*i*_(*k*) the portion of the total investment assigned to an asset *i* at step *k*, for *i* = 1…, *p*, and *k* = 1, …, *T*. In vector notation, the portfolio **x** at period *k* is built as

$$\begin{array}{c} x\left(k\right)=(x_{1}\left(k\right),\ldots ,x_{p}\left(k\right)). \end{array}$$ (1)

In the same way, the total wealth at period *k* is given by

$$\begin{array}{c} W\left(k\right)=\sum_{i=1}^{p}x_{i}\left(k\right)=1^{\prime }x\left(k\right). \end{array}$$ (2)

Suppose that at the end of each period, the portfolio is rebalanced. Let us denote by **x**^+^(*k*) the adjusted portfolio at period *k* with optimized variable **u**(*k*)

$$\begin{array}{c} x^{+}\left(k\right)=x\left(k\right)+u\left(k\right). \end{array}$$ (3)

Further, let us assume the portfolio is self financing, i.e., no cash is injected into it or withdrawn from it. Then,

$$\begin{array}{c} \sum_{i}^{p}u_{i}\left(k\right)=0. \end{array}$$ (4)

On the other hand, the profit of asset *i* at time *k* is given by

$$\begin{array}{c} g_{i}\left(k\right)=r_{i}\left(k\right)+1, \end{array}$$ (5)

where *r*_*i*_(*k*) is the return of asset *i* at time *k*. If we construct the diagonal matrix **G**(1) = *diag*(**g**_1_(1), …, **g**_*p*_(1)), the portfolio composition at the end of the first period can be written as

$$\begin{array}{c} x(1)=G(1)x(0)+G(1)u(0). \end{array}$$ (6)

Then, the dynamic equation of the portfolio at the end of the period *k* + 1 is given by

$$\begin{array}{c} x(k+1)=G(k+1)x(k)+G(k+1)u(k),\;k=0,\ldots ,t-1. \end{array}$$ (7)

Using this recursion formula, the total investment capital at step *k* can be expressed as

$$\begin{array}{c} W\left(k\right)=1^{\prime }x\left(k\right)=\Phi^{\prime }\left(1,k\right)x\left(0\right)+\sum_{j=1}^{k}\Phi^{\prime }\left(j,k\right)u\left(j-1\right), \end{array}$$ (8)

where **Φ**′(*j*, *k*) = 1′**G**(*k*)**G**(*k* − 1)…**G**(*j*), y **Φ**′(*k*, *k*) = 1′**G**(*k*). Hence, total investment risk is quantified as a weighted sum of capital volatility during the *k* stages

$$\begin{array}{c} J\left(t\right)=\sum_{k=1}^{t}\gamma \left(k\right)\text{var}\left\{W\left(k\right)\right\}, \end{array}$$ (9)

where *γ*(*k*) ≥ 0 is the level of risk associated with each period, which is set heuristically.

In this multiperiod problem, the objective is to minimize the risk at the end of the investment trajectory, represented by *J*(*T*), while satisfying a lower bound constraint on the total return **Φ**_*lb*_. The quantity **Φ**_*lb*_ is directly related to the minimum bound of the desired replacement rate of the investment fund. In practice, an optimal frontier is obtained for a set of desired replacement rates accomplished with the lowest total investment risk in a range of values. Thus, we obtain a set of optimal solutions, each with a particular glide-path. Then, we can choose a solution that meets the particular risk aversion of the investor, similar to the optimal frontier of Markowitz [10]. Note that *J*(*t*) is the objective function that we will seek to optimize under the specific investment limits or restrictions listed in Table 2 applied to the assets of Table 1.

**Table 1:** Assets considered to this study.

Category	Name	Asset	Identifier
c1	Structured	BBVA B+EST1	STRUCTURED
c2	Commodities	SP’GSCITR S&P GSCI Total Return Index	COMMODITIES
c3	Fibras	Fibra Uno Administracion (FUNO11)	FIBRAS
c4	Equities	MSCI All Country World Index S&P/BMV IPC	ACWI IPC
c5	Securitized	CEMEX (CEMEXCPO) Fomento Economico Mexicano (FEMSAUBD)	CEMEX FEMSA
c6	Foreign Assets	Global-Aggregate Total Return Index MSCI World Real Estate Index	GAI REITS
c7	Udibonos (lower)	SPVIFUBT Index	UDIBONOS
c8	FOREX	USD-MXN X-RATE U.S. Dollar Index	MXN DXY
c9	Unbounded	S&P/BMV Sovereign MBONOS Bond Index 182-day CETES	MBONES CETES182

The first column enumerates the asset’s category. The second column lists the names of categories. The third column describes the full name of each asset. The fourth column represents the assets by an identifier.

### 2.2 Model as a convex quadratic programming problem

In [12] the convexity of *J*(*t*) under the assumptions discussed above is proved. Explicitly, the objective function is minimized at each stage *k* by the model

$$\begin{array}{c} \begin{array}{cc} & \underset{\bar{u}\left(0\right),\ldots ,\bar{u}\left(T-1\right)}{min}\sum_{k=1}^{T}\gamma \left(k\right)\text{var}\left\{W\left(k\right)\right\}\\ & \text{subjet}\;\text{to}:\\ & E\left\{W\left(T\right)\right\}\geq \Phi_{lb}x\left(0\right),\\ & 1^{\prime }\bar{u}\left(k\right)=0,\;k=0,\ldots ,T-1\\ & E\{x^{+}\left(k\right)\}\geq 0,k=0,\ldots ,T-1\\ & \nu_{lb,j}\left(k\right)1^{\prime }E\left\{x\left(k\right)\right\}\leq \sum_{i\in c_{j}}(E\left\{x_{i}\left(k\right)\right\}+{\bar{u}}_{i}\left(k\right))\leq \nu_{ub,j}\left(k\right)1^{\prime }E\left\{x\left(k\right)\right\},\\ & j=1,\ldots ,l;k=0,\ldots ,T-1 \end{array} \end{array}$$ (10)

where:

- $\bar{u}\left(j\right)$: Optimal expected adjustment vector at step *j*. This provides the optimal portfolio rebalancing at the end of each stage as a result of optimizing the objective function.
- **Φ**_*lb*_: Lower bound of the portfolio return at the end of the period. It is associated with the replacement rate.
- *ν*_*lb*,*i*_,*ν*_*up*,*i*_: lower and upper bound, respectively, of the sum of asset weights in category *c*_*j*_, where *j* = 1, …, *l*. This restriction allows us to limit, for example, the proportion of high- and low-risk assets to add to the portfolio. In our case, we have *l* = 9 categories of different restrictions.
- *γ*(*k*): These values weight the risk contribution of each stage in the global investment trajectory.

Furthermore, the values of *E*{*W*(*k*)}, *E*{**x**(*k*)} and *var*{*W*(*k*)} are estimated as a function of the optimal values of $\bar{u}$ as well as through the estimated covariance matrix of the model and the expected returns.

In practice, the investment in a fund is not expected to suffer significant changes in two consecutive periods. For this reason, we add the constraints (11) in order to control the adjustment performed at each period.

$$\begin{array}{c} -\alpha W\left(k\right)\leq {\bar{u}}_{i}\left(k\right)\leq \alpha W\left(k\right)\;\forall \;k=1,\ldots ,T;\;i=1,\ldots ,p; \end{array}$$ (11)

where *α* indicates the maximum and the minimum portion of adjustment allowed from the total wealth at each period.

## 3 Volatility models

A way to estimate the expected returns and covariance matrices needed to optimize (10) is through the analysis of volatility. As explained by [13] among many others, financial time series exhibit a series of statistical regularities which successful models necessarily take into account. In particular, returns are unpredictable, have large and frequent outliers, outliers in both directions tend to be clustered in time, and returns may have an asymmetric impact on volatility.

The main way to incorporate these features into a time series model is to use a multiplicative model where returns are expressed in terms of the unobservable volatility process, namely:

$$\begin{array}{c} \varepsilon_{t}=\sigma_{t}Z_{t}, \end{array}$$

where the random process {*Z*_*t*_} is white noise and the volatility process $\left\{\sigma_{t}^{2}\right\}$ models, dynamically, the conditional variance of the returns given past information. Two alternative specifications have been suggested in the literature for the volatility process: the GARCH (Generalized Autorregressive Conditional Heteroskedasticity) family and the ARSV (Autorregressive Stochastic Volatility) models.

GARCH models were first introduced in [14, 15] and specify *σ*_*t*_ as a function of a vector of unknown parameters *θ*. Due to its success in several fields of application, and in order to better accommodate different features of financial data, the original GARCH model has seen a number of extensions and modifications. Most of these extensions are included in the f-GARCH model, family GARCH, of [16] according to which *θ* = (*α*, *β*, λ, *ν*, *b*, *c*)^*T*^, and

$$\begin{array}{c} \begin{array}{cc} \frac{\sigma_{t}^{\lambda }-1}{\lambda }= & \;\omega +\sum_{i=1}^{q}\alpha_{i}\sigma_{t-i}f{\left(\varepsilon_{t+i-1}\right)}^{\nu }+\sum_{j=1}^{p}\beta_{j}\frac{\sigma_{t-j}^{\lambda }-1}{\lambda },\\ f(\varepsilon_{t})= & \;|\varepsilon_{t}-b|-c\left(\varepsilon_{t}-b\right). \end{array} \end{array}$$ (12)

Different choices of the orders *p*, *q* and the parameters in *θ* provide different models. Notice that the volatility process *σ*_*t*_ is not directly modeled; but is instead included via a Box-Cox transformation. The first parameter, λ, governs the shape of this transformation. As customary, we take the Box-Cox transformation with λ = 0 as the logartithmic transformation. The parameters {*α*_*i*_, *i* = …, *q*} capture the impact of past returns on the variance. In this sense, they are considered the ARCH part of the model. The influence of past returns of the volatily process is modulated by the function *f* and the power parameter *ν*. The main reason to include *f* in this formulation is to allow for asymmetric responses so that negative and positive returns have a different impact on the volatility. This asymmetry, also known as leverage, is an important part of financial theory. Parameters *a*, *b*, *c*, and *ν* allow for a flexible specification of the asymmetric effect. Finally, the parameters {*β*_*j*_, *j* = 1…, *p*}, known as the GARCH part of the model, measure the influence of past volatilities on the present one.

Stochastic Volatility Models, on the other hand, can be traced to the framework of [17] and model the dynamics of the volatility by introducing a stochastic process directly into it. One of the most representative models in this family is the Autorregressive Stochastic Volatility Model (ARSV) process which specifies

$$\begin{array}{c} \begin{array}{c} \sigma_{t}=\sigma \text{exp}\left\{h_{t}/2\right\},\\ h_{t}=\phi h_{t-1}+\eta_{t}. \end{array} \end{array}$$

The stochastic process *η*_*t*_ is white noise, usually Gaussian, and independent of *Z*_*t*_. The stochastic properties of the model, which make it suitable for modeling financial series, can be found in [18–20]. Just as in GARCH models, asymmetric effects have been incorporated into the SV framework by different authors as early as [21, 22] and later revisited by [23]

Further generalizations of this asymmetric response which simultaneously add a strong non-linear effect have been given which are inspired in the Threshold Autorregressive Model of [24, 25]. Threshold non-linearity is incorporated into the mean and variance specifications of a SV model by [26] in his Threshold Stochastic Volatility model (THSV) through

$$\begin{array}{c} \text{log}\;\sigma_{t+1}^{2}=\alpha_{S_{t+1}}+\phi_{S_{t+1}}\text{log}\left(\sigma_{t}^{2}\right)+\eta_{t}, \end{array}$$ (13)

In this equation, *S*_*t*_ is the indicator function of *ε*_*t*_ ≥ 0 so that

$$\begin{array}{c} S_{t}=\{\begin{array}{cc} 1, & \varepsilon_{t}\geq 0,\\ 0, & \varepsilon_{t}<0. \end{array} \end{array}$$

Therefore, the parameters in the volatility process have two states which depend on the sign of past returns. Parameter *ϕ* takes on value, say, *ϕ*_1_ if *S*_*t*_ = 1 and value *ϕ*_2_ if *S*_*t*_ = 0. A very similar model, which uses Eq (13) but omits the term $\alpha_{S_{t}}$ and only deals with asymmetry in the variance specification, was introduced some years later by [27] and labeled Threshold Asymmetric Autorregressive SV (TA-ARSV) model. Later, in [28] the TA-ARSV is again put forward as a modelling strategy and applied to precious metal returns. According to the authors, and based on a selected sample of returns of financial stock and three precious metals, the TA-ARSV is more sensitive to asymmetries and more capable of measuring the leverage effect when compared to the A-GARCH, T-GARCH, A-ARSV, and ARSV models. Like GARCH processes, threshold SV models have been applied in several fields and capture the statistical regularities of different financial instruments and price processes. For example, the leverage effect of spot prices in the energy market have been studied in [29, 30]. The model was also successfully applied to stock markets in [27, 31, 32], to daily average prices of energy products in [33], and to predict crypto and wold currencies in [34].

While all these models may be appropriate for modelling certain forms of the leverage effect in volatility, they offer no distinct advantage over the f-GARCH asymmetric models for our modelling purposes. In this paper we focus thus on returns generated by Eq (12) and seek to optimize the investment trajectories based on them. Different approaches to portfolio optimization and hedging using GARCH type models and asymmetric correlations can be found in, for example, [35, 36].

We will further specify that *p* = *q* = 1, in which case both, *α* and *β* are only real numbers. Most of applied work is done around this specification, although some authors argue that other values for *p* and *q* could be more appropriate. See, for example, section 8.5 of [37]. Under this assumption, if λ = 0, *ν* = 1, *b* = 0 we obtain the exponential GARCH(1, 1) model of [38], whereas if λ = *ν* = 2, *b* = 0 we obtain the GJR-GARCH(1, 1) of [39] which are particularly useful in financial applications due to their ability to measure the leverage effect. The reader is referred to [16, 37, 40, 41] for a survey of GARCH models and some of their uses in finance.

Several generalizations to multivariate models for conditional heteroskedasticity, M-GARCH for short, have been proposed in the literature. Generally speaking, the data is now modeled as a random vector **x**_*t*_ = (*x*_1,*t*_, …, *x*_*n*,*t*_)′ with joint dynamics given by

$$\begin{array}{cc} x_{t} & =\mu_{t}\left(\theta \right)+\epsilon_{t}, \end{array}$$ (14)

$$\begin{array}{cc} \epsilon_{t} & =\Sigma_{t}^{1/2}\left(\theta \right)z_{t}, \end{array}$$ (15)

where *μ*_*t*_(*θ*) is the *n* × 1 vector of conditional means, **Σ**_*t*_(*θ*)^1/2^ denotes a squared root of the *n* × *n* conditional covariance matrix of the vector **ϵ**_*t*_, and *θ* is a vector of unknown parameters. M-GARCH models can be roughly classified as either direct generalizations of the univariate GARCH (VEC, BEKK, and Factor Models), weighted averages of univariate GARCH models (O-GARCH, GO-GARCH), or nonlinear transformations mixing univariate GARCH models (Dynamic covariance GARCH models such as CCC, DCC, GDC, and Copula GARCH). Each of these models admits a further generalization to incorporate leverage or asymmetry. For a full review of M-GARCH models, the reader is referred to [42].

In this paper, we forecast the expected returns and the covariance matrix of the portfolio of Table 1 using two of the asymmetric specifications of the univariate GARCH model, namely, the EGARCH of [38] and the GJR-GARCH of [39], and estimate the conditional covariance matrix of such returns using the Dynamic Conditional Correlations M-GARCH. More specifically, we let the covariance matrix **Σ**_*t*_ be factored as

$$\begin{array}{c} \Sigma_{t}=D_{t}^{1/2}R_{t}D_{t}^{1/2}, \end{array}$$ (16)

where *D*_*t*_ is *n* × *n* the diagonal matrix of conditional variances and **R**_**t**_ is the *n* × *n* conditional correlation matrix. One great advantage of this model is that it greatly reduces the number of parameters to estimate without imposing serious restrictions on the parameter space. Follwing [43], we further let

$$\begin{array}{cc} & R_{t}=diag{\left(Q_{t}\right)}^{-1/2}\times Q_{t}\times diag{\left(Q_{t}\right)}^{-1/2} \end{array}$$ (17)

$$\begin{array}{cc} & Q_{t}=\left(1-\delta_{1}-\delta_{2}\right)\bar{Q}+\delta_{1}\left(u_{t-1}u_{t-1}^{\prime }\right)+\delta_{2}Q_{t-1}, \end{array}$$ (18)

where $\bar{Q}$ is the unconditional covariance matrix of *u*_*t*_ = {*ϵ*_*i*,*t*_/*σ*_*i*,*t*_}_*i*=1,…,*n*_ and

$$\begin{array}{cc} & 0\leq \delta_{1},\delta_{2}\leq 1, \end{array}$$ (19)

$$\begin{array}{cc} & \delta_{1}+\delta_{2}\leq 1, \end{array}$$ (20)

is assumed, which guarantees the positive definitiveness of **R**_*t*_.

## 4 Transfer entropy

In order to evaluate whether the dependencies between the variables are preserved before and after applying the volatility models to the assets under study, we have measured the Transfer Entropy (TE). Next, we draw the main elements of the theory.

Let *x*_*i*_ = *x*(*i*) and *y*_*i*_ = *y*(*i*), *i* = 1, …, *N*; denote a series of observations of systems *X* and *Y*. TE measure is defined as [44]

$$\begin{array}{c} T_{Y\to X}\left(k,l\right)=\sum_{i,j}p\left(x_{t+1},x_{t}^{\left(k\right)},y_{t}^{\left(l\right)}\right)\text{log}\frac{p(x_{t+1}|x_{t}^{\left(k\right)},y_{t}^{\left(l\right)})}{p(x_{t+1}|x_{t}^{\left(k\right)})}, \end{array}$$ (21)

TE attempts to incorporate time dependence into account by relating previous observations *x*_*i*_ and *y*_*i*_ in order to predict the next value *x*_*i*+1_. Then, it quantifies the deviation from the generalized Markov property, *p*(*x*_*i*+1_|*x*_*i*_, *y*_*i*_) = *p*(*x*_*i*+1_|*x*_*i*_), where *p* denotes the transition probability density to state *x*_*i*+1_ given *x*_*i*_ and *y*_*i*_. If there is no deviation from the generalized Markov property, *Y* has no influence on *X*. Then, TE quantifies the incorrectness of this assumption, and being formulated as the Kullback-Leibler entropy between *p*(*x*_*i*+1_|*x*_*i*_, *y*_*i*_) and *p*(*x*_*i*+1_|*x*_*i*_) is explicitly nonsymmetric with respect to the exchange of *x*_*i*_ and *y*_*i*_.

A straightforward approach to estimate TE is to partition the data into discretized values. Thus, a time series *x*(*t*) is partitioned as follows to obtain the symbolically encoded sequence *S*(*t*)

$$\begin{array}{c} S_{t}=\{\begin{array}{cc} 1 & \text{for}\;y_{t}\leq q_{1}\\ 2 & \text{for}\;q_{1}<y_{t}\leq q_{2}\\ \vdots & \vdots \\ n-1 & \text{for}\;q_{n-1}<y_{t}<q_{n}\\ n & \text{for}\;q_{n} \end{array} \end{array}$$ (22)

The above symbolic sequence replaces the value in the observed time series by the discrete states {1, 2, …, *n* − 1, *n*}.

Nevertheless, the expression of TE is likely to be biased due to several factors such as finite sample effects and the not strict stationarity of financial data. Also, time series with higher entropy naturally transfer more entropy to the others. To reduce this bias the Effective Transfer Entropy (ETE) was proposed in [45] and defined as

$$\begin{array}{c} ETE_{Y\to X}^{shuffled}\left(k,l\right):T_{Y\to X}\left(k,l\right)-T_{Y_{shuffled}\to X}\left(k,l\right), \end{array}$$ (23)

where *T*_*Y*_*shuffled*_→*X*_ indicates the transfer entropy from *Y* to *X* with randomly shuffled time series *Y*. Thus, all statistical dependencies between the two time series are destroyed. An important characteristic is that *T*_*Y*_*shuffled*_→*X*_(*k*, *l*) converges to zero at long sample size. Consequently, any non-zero value of *T*_*Y*_*shuffled*_→*X*_(*k*, *l*) is due to small sample effects.

The work of Dimpfl et. al. [46] improves the bias correction by adding an inferencial perspective to the estimated information flows. They proposed to use the Horowitz’s approach [47], who bootstraps the modelled Markov process. The idea is to simulate process *Y* based on the calculated transition probabilities, where the dependencies between *Y* and *X* are destroyed, but the dynamics of the series *Y* is not changed. Transfer entropy is then estimated using the simulated time series. Then, this procedure is repeated several times to create a null distribution of no information flow, which can be used to test for statistical significance. The proposed equation has the same structure as Eq 23:

$$\begin{array}{c} ETE_{Y\to X}^{boot}\left(k,l\right):T_{Y\to X}\left(k,l\right)-T_{Y_{boot}\to X}\left(k,l\right), \end{array}$$ (24)

where *T*_*Y*_*boot*_→*X*_ indicates the average over the estimates derived from the null bootstrap distribution.

## 5 Data

We consider weekly prices of 14 instruments available in the Mexican Stock Exchange (BMV, for its acronym in Spanish) from September 21st 2012 to February 14th 2020 for a total of *n* = 386 trading weeks (see 8). The data were accessed from Bloomberg (Available at https://www.bloomberg.com/) and Yahoo Finance (Available at https://finance.yahoo.com/). This period includes the most recent shocks in the Mexican capital markets as are the uncertainty of the FX, the fluctuations of debt and equity market due to the American and Mexican elections. It also includes the NAFTA renegotiations in the 2016–2018 period [5], but does not take into account the recent financial crash resulting from COVID-19.

The weekly frequency is chosen to have enough observations to avoid bias in the estimation and, in part, because it was not possible to go beyond 2012 in the past for some of the selected assets. Also, we avoid higher resolution data (daily, for example) because forecasting becomes much more complex and computationally demanding. The data thus gathered presents less than 1% of missing values, which were imputed using splines interpolation of order three.

The assets are listed in Table 1, where the first column shows the asset’s category with respect to the allowed weight under the portfolio strategy explained in the next section. The second column presents the name of the categories. The full name of assets belonging to each category are listed in the third column, and the fourth column describes each asset by an identifier. This particular selection is inspired by the typical practitioner preferences of fund administrators and in accordance with the new Mexican regulation [7]. In our asset selection, we take into account the empirical evidence that suggests a well-diversified portfolio should include a healthy allocation to international equities [48].

In addition, Table 2 presents the investment limits proposed in this study. The 161 quarters periods were annualized in order to reduce the computational complexity of the multistage allocation problem. Nonetheless they are established according to the new regulation of the SAR agency in Mexico, where all the columns represent the upper limits allowable under the regulation, except for category *c*7, which represents a lower limit.

**Table 2:** Investment limits considered for each category.

Period	c1	c2	c3	c4	c5	c6	c7	c8	c9
1	20.00	5.00	10.00	59.68	39.73	20.00	51.00	30.00	100.00
2	20.00	5.00	10.00	59.27	39.43	20.00	51.00	30.00	100.00
3	20.00	5.00	10.00	58.90	39.08	20.00	51.00	30.00	100.00
4	20.00	5.00	10.00	58.57	38.70	20.00	51.00	30.00	100.00
5	20.00	5.00	10.00	58.22	38.28	20.00	51.00	30.00	100.00
6	20.00	5.00	10.00	57.83	37.84	20.00	51.00	30.00	100.00
7	20.00	5.00	10.00	57.41	37.37	20.00	51.00	30.00	100.00
8	20.00	5.00	10.00	56.96	36.87	20.00	51.00	30.00	100.00
9	20.00	5.00	10.00	56.48	36.35	20.00	51.00	30.00	100.00
10	20.00	5.00	10.00	55.97	35.82	20.00	51.00	30.00	100.00
11	20.00	5.00	10.00	55.45	35.27	20.00	51.00	30.00	100.00
12	20.00	5.00	10.00	54.89	34.71	20.00	51.00	30.00	100.00
13	20.00	5.00	10.00	54.32	34.14	20.00	51.00	30.00	100.00
14	20.00	5.00	10.00	53.72	33.57	20.00	51.00	30.00	100.00
15	20.00	5.00	10.00	53.11	32.99	20.00	51.00	30.00	100.00
16	20.00	5.00	10.00	52.48	32.41	20.00	51.00	30.00	100.00
17	20.00	5.00	10.00	51.83	31.83	20.00	51.00	30.00	100.00
18	20.00	5.00	10.00	51.16	31.26	20.00	51.00	30.00	100.00
19	20.00	5.00	10.00	50.40	30.70	20.00	51.00	30.00	100.00
20	20.00	5.00	10.00	49.38	30.16	20.00	51.00	30.00	100.00
21	19.43	5.00	9.71	48.30	29.58	20.00	51.00	30.00	100.00
22	18.86	5.00	9.43	47.15	28.95	20.00	51.00	30.00	100.00
23	18.29	5.00	9.14	45.93	28.26	20.00	51.00	30.00	100.00
24	17.71	5.00	8.86	44.66	27.47	20.00	51.00	30.00	100.00
25	17.14	5.00	8.57	43.33	26.66	20.00	51.00	30.00	100.00
26	16.57	5.00	8.29	41.90	25.85	20.00	51.00	30.00	100.00
27	16.00	5.00	8.00	40.30	25.00	20.00	51.00	30.00	100.00
28	15.43	5.00	7.71	38.51	24.30	20.00	51.00	30.00	100.00
29	14.86	5.00	7.43	36.48	23.70	20.00	51.00	30.00	100.00
30	14.29	5.00	7.14	34.15	23.12	20.00	51.00	30.00	100.00
31	13.71	5.00	6.86	31.48	22.56	20.00	51.00	30.00	100.00
32	13.14	5.00	6.57	28.42	22.04	20.00	51.00	30.00	100.00
33	12.57	5.00	6.29	25.10	21.56	20.00	51.00	30.00	100.00
34	12.00	5.00	6.00	21.82	21.12	20.00	51.00	30.00	100.00
35	11.43	5.00	5.71	18.88	20.73	20.00	51.00	30.00	100.00
36	10.86	5.00	5.43	16.58	20.39	20.00	51.00	30.00	100.00
37	10.29	5.00	5.14	15.23	20.11	20.00	51.00	30.00	100.00
38	10.00	5.00	5.00	15.00	20.00	20.00	51.00	30.00	100.00
39	10.00	5.00	5.00	15.00	20.00	20.00	51.00	30.00	100.00
40	10.00	5.00	5.00	15.00	20.00	20.00	51.00	30.00	100.00

All the cases represent an upper limit, except for *c*7, which fixes a lower limit.

## 6 Expected returns and covariance matrices

The EGARCH(1,1), GJR-GARCH(1,1), GARCH(1,1) are considered to estimate the expected returns and covariance matrices. The methodology is described below and implemented on R using the libraries rugarch and rmgarch:

(a) For each historical price time series of assets, the difference in logarithmic values is calculated to obtain the returns. This transformation helps to eliminate the trend component and transforms the time series into its stationary forms.
(b) The EGARCH, GJR-GARCH, and GARCH models of order (1,1) with constant mean and normal distribution of residuals are fitted to the empirical returns. The three approaches are fitted independently to compare the robustness of the glide-path under the different models.
(c) To fit the model is followed a hybrid strategy in the sense that as a first attempt is used the solver solnp proposed in [49]. If the solver does not converge, then it is used one of the following solvers of the rugarch library: nlminb, gosolnp, nloptr; which priority is given in the same order [50].
(d) Given the prediction horizon *H* and the number of desired replications, *B*, we generate *B* bootstrap replications of the adjusted process with a path length of *H*. These trajectories can be interpreted as *B* possible scenarios for the next *H* weeks. In our specifications, we choose *B* = 1000 and *H* = 2093 weeks, the last because we are interested in studying the standard horizon of a glide-path of 161 quarters, where each quarter is composed of 13 weeks.
(e) For the EGARCH and GJR-GARCH, the B trajectories of the bootstrap replications are estimated by sampling with replacement in a semi-parametric approach. In the first step it is obtained an estimation of the standard deviation ${\hat{\sigma }}_{t}$ using innovations *z*_*t*_ ∼ *N*(0, 1) and the empirical residuals *ϵ*_*t*_. Then, in a second step we adjust the innovations ${\hat{z}}_{t}=\epsilon_{t}{\hat{\sigma }}_{t}$. In this way, the normal distribution specification for the innovations is replaced by the non-parametric adjusted distribution of the empirical data. In the GARCH case, we followed a parametric approach with *z*_*t*_ ∼ *N*(0, 1).
(f) The covariance matrix is estimated under the DCC-GARCH(1,1) at each period *k* = 1, …, *T*; and for each univariate model: EGARCH(1,1), GJR-GARCH(1,1), and GARCH(1,1). Here, we consider a jointly Gaussian distribution and a Generalized Error Distribution (GED) for the univariate specifications of innovations.
(g) The estimated weekly expected returns are aggregated to obtain the annual expected returns.
  $$\begin{array}{c} r_{i}^{\left(annual\right)}\left(k\right)=\sum_{j=1}^{Y}r_{i}\left(j+\left(k-1\right)Y\right), \end{array}$$ (25)
  for period *k* = 1, …, *T* = 40, and asset *i* = 1, …, *p* = 14. Similarly, the annual covariance matrix is obtained by multiplying the weekly results by a factor of *Y* = 52. This is done under the assumption of an underlying random walk process in the dynamics of returns.
(h) As a baseline comparison, we consider a Stationary Block Bootstrap Simulation (SBBS) for the future trajectory of the set of assets following the idea of [51]. In this approach, the block size follows a geometric distribution, where we set the expected value as *μ* = 13; the number of weeks that compose a quarter. As an equivalent of expected returns, we consider the mean of *B* = 1000 replications of size *H* = 2093 for each asset. Finally, the aggregated annual expected returns are computed in the same way as the previous step. In this case, we do not estimate the covariance matrix.

**Fig 1:**
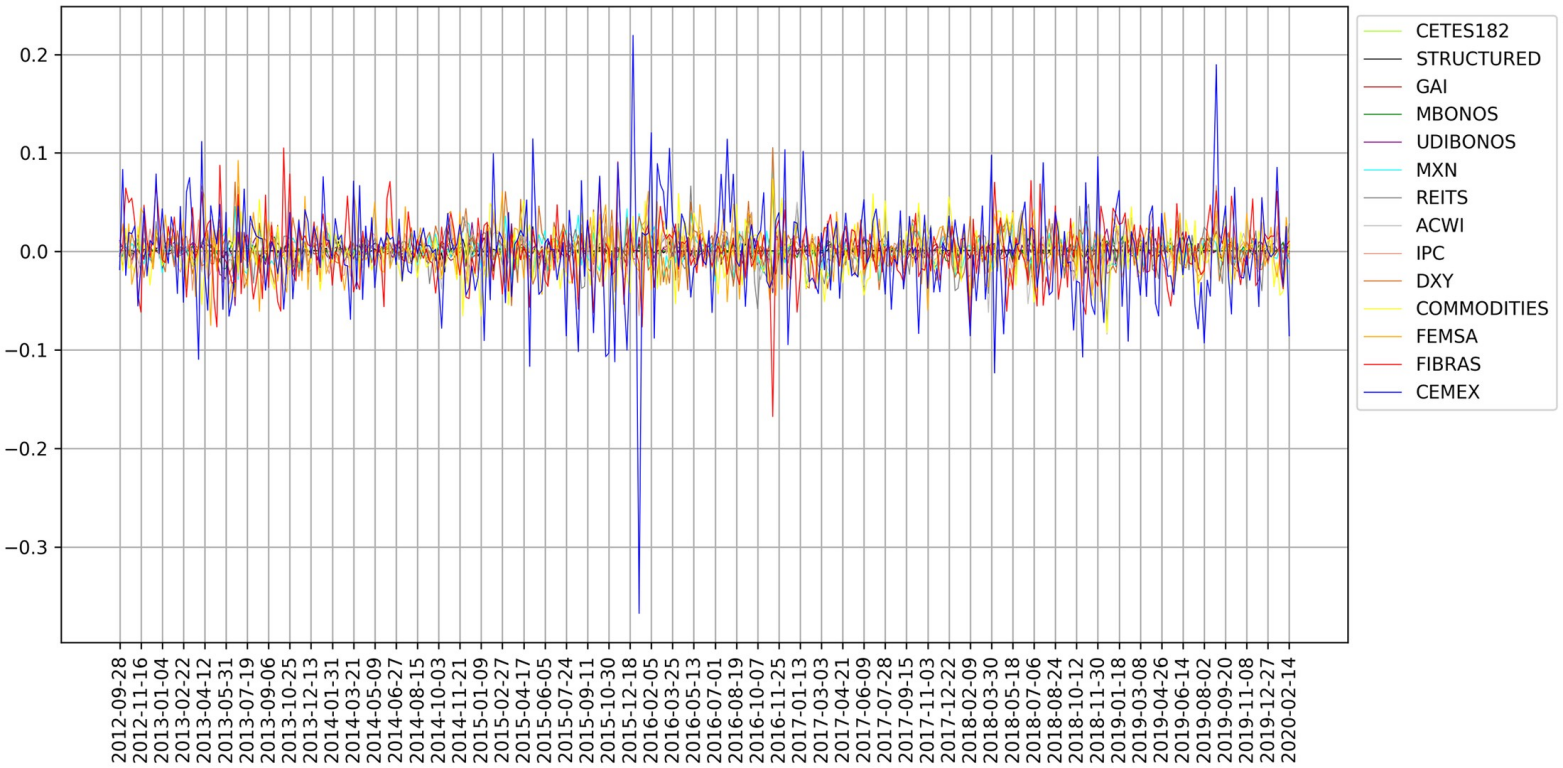
Historical returns.

The historical price time series of assets listed in Table 1 is preprocessed according to the methodology described above and the weekly historical returns are obtained, which are shown in Fig 1. These returns are the input for the forecast model for each of the *p* = 14 assets.

**Fig 2:**
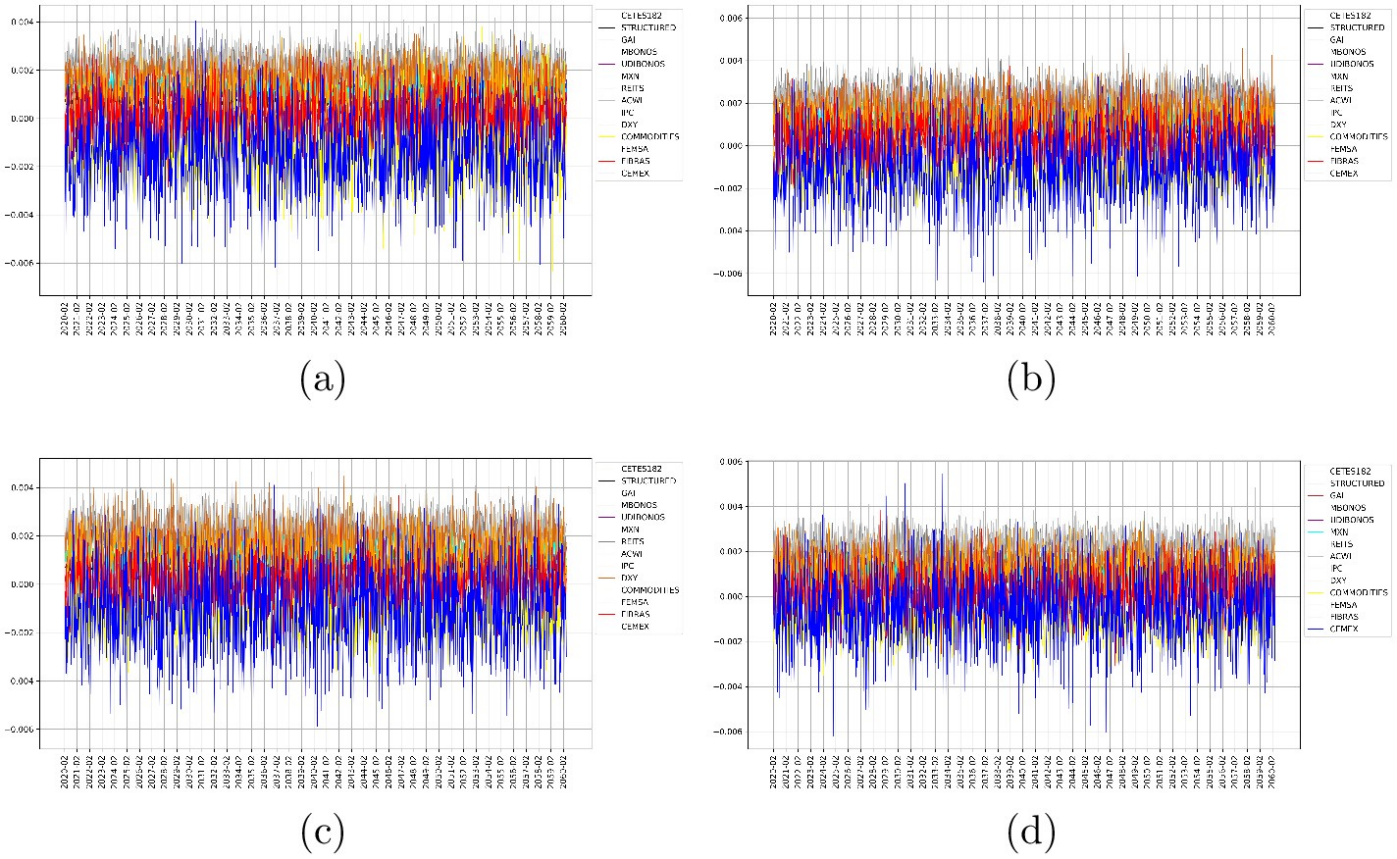
Forecast of expected returns. (a) Figure at top left: results obtained with EGARCH models. (b) Figure at top right: results obtained with the GJR model. (c) Figure at bottom left: results obtained with the GARCH model. (d) Figure at bottom right: results obtained with SBBS model.

**Fig 3:**
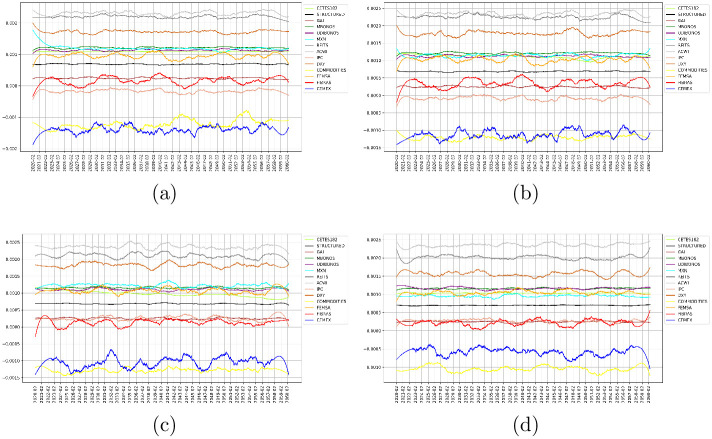
Filtered expected returns. (a) Figure at top left: results obtained with EGARCH models. (b) Figure at top right: results obtained with the GJR model. (c) Figure at bottom left: results obtained with the GARCH model. (d) Figure at bottom right: results obtained with SBBS model.

The weekly expected returns for the whole forecasting period are shown in Fig 2. It can be seen that estimated expected returns are two orders of magnitude below historical returns. Hence, in agreement with GARCH theory in the sense that the mean or expected value converges to zero. Further, we annualized the returns and applied the Savitsky-Golay filter [52] to yield smoothed input values for the objective function of the multistage portfolio optimization. The filtered expected return series are shown in Fig 3, where we have chosen a 5-year moving window and a polynomial of order three with interpolated extension to the padded signal. We can notice the volatility is reduced drastically, which in some sense can be interpreted as the stochastic nature has been omitted and then we preserve only the deterministic trend. At a simple glance, it can be noticed that the volatility and scale of the expected returns do not vary across the different forecasting strategies followed.

**Fig 4:**
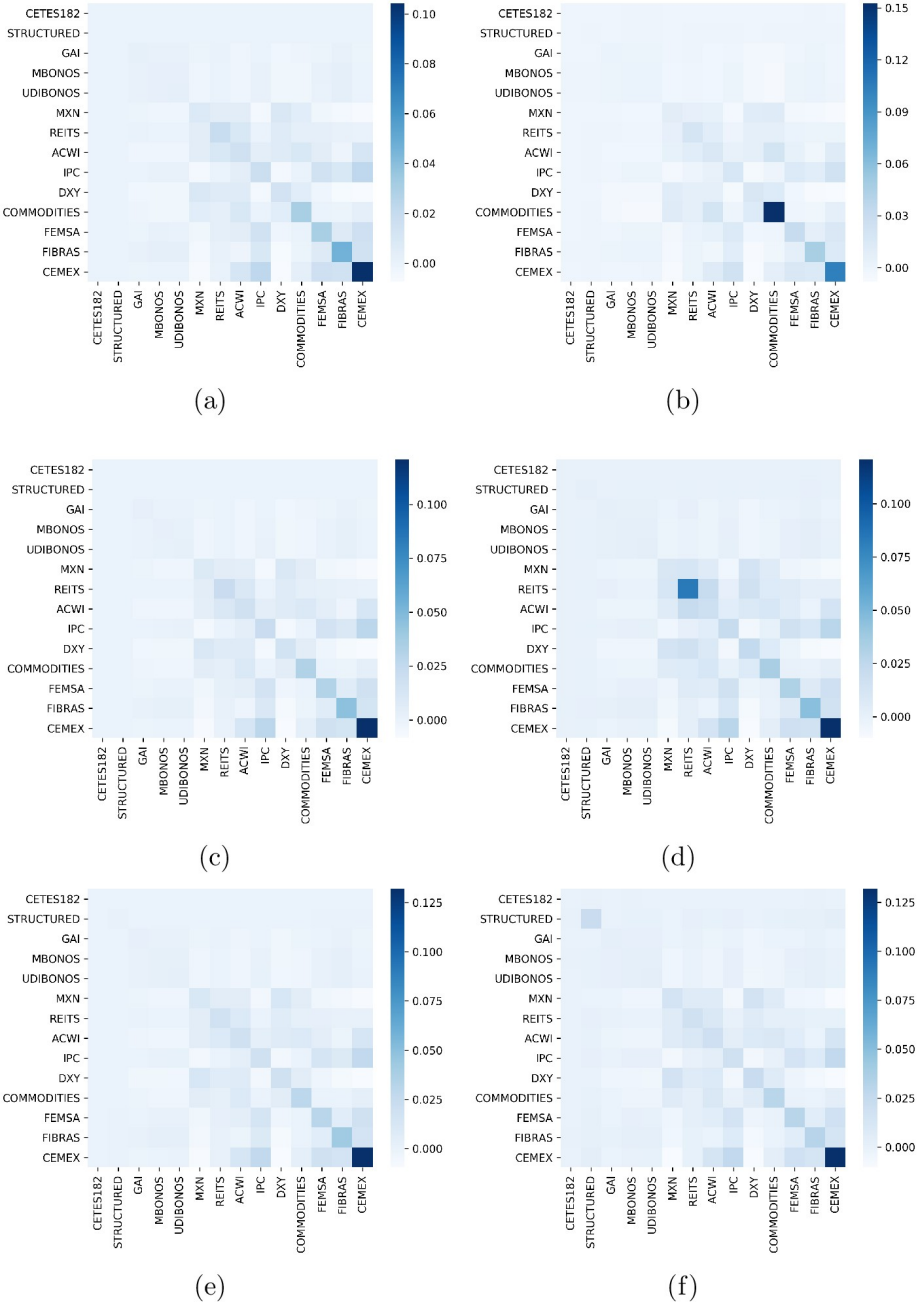
Estimated covariance matrices under DCC-GARCH(1,1). Top, univariate EGARCH specification at *t* = 1, 40 years ((a) and (b), respectively). Middle, univariate GJR-GARCH specification at *t* = 1, 40 years ((c) and (d), respectively). Bottom, univariate GARCH specification at *t* = 1, 40 years ((e) and (f), respectively).

As described above, the covariance matrix was estimated under the DCC-GARCH(1,1). To illustrate the quality of the results, Fig 4 plot heatmaps of the historical and estimated covariance matrices at year = 1 and 40; obtained using the univariate specifications given by the EGARCH(1,1) and GJR-GARCH(1,1) and GARCH(1,1) models, respectively. In the same figure, the assets are organized from lower to higher standard deviation according to their historical values. Here, can be seen a block structure of the covariance matrices, where the pattern does not change significantly over time. The most distinctive block is composed of MXN, REITS, ACWI, IPC, DXY, COMMODITIES, FEMSA, FIBRAS, and CEMEX. These assets belong to the categories of FOREX, Foreign Assets, Securitized, Equities, Fibras, and Commodities, which are more volatile by their nature. Then, a clear separation between bonds and risky assets stands out in this representation.

### 6.1 Asymmetric dependencies

The asymmetric dependencies between assets were assessed by the Transfer Entropy measure as explained in section 4. In particular, we compute ETE for the set of historical returns and for each set of the estimated expected returns obtained by the different models. The estimation of ETE was done considering a Markov order *k* = *l* = 1 and 300 bootstrap replications for each direction of the estimated transfer entropy. Moreover, we drop the 50 first observations of the Markov chain in each bootstrap simulation to avoid transitory effects, and set the number of shuffles as 100.

**Fig 5:**
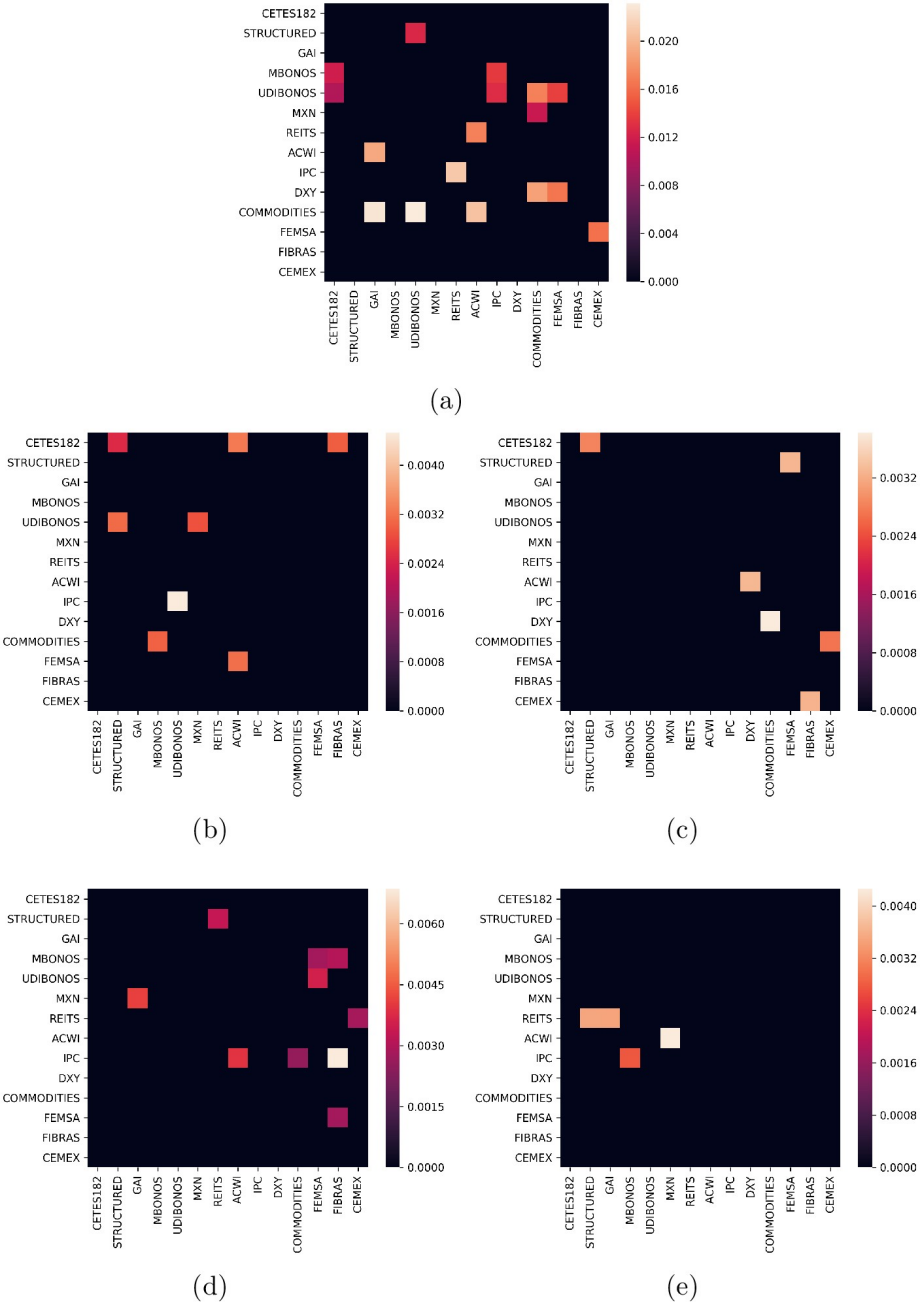
Effective transfer entropy. (a) Heatmap of ETE over historical returns. Heatmap of ETE over expected returns estimated by (b) EGARCH(1,1), (c) GJR-GARCH(1,1), (d) GARCH(1,1), and (e) SBBS model. In all cases the Markov order is set as *k* = *l* = 1. The number of bootstrap replications for each direction of the estimated transfer entropy is chosen as *B* = 300 with 100 shuffles. The first 50 observations of the bootstrapped Markov are removed.

We can see in Fig 5 the heatmaps of ETE (Eq 23), in units of bits, from the assets of the vertical axis to the asset on the horizontal axis. Here, it is plotted only the values that are statistically significant, specifically, satisfying the condition of having a p-value<0.05 when testing against the empirical bootstrap distribution. For example, we can notice in Fig 5(a) a statistically significant flow of information from DXY to COMMODITIES but not vice versa. Only in the expected returns forecasted by GJR-GARCH(1,1) model this direct flow of information is preserved, whereas the majority of the other links are not correctly captured.

We measure the True Positive Rate (TPR) and the True Negative Rate (TNR) in order to quantify the number of asymmetric dependencies that are preserved on the future trajectory of each asset under the different model. These quantities are expressed as

$$\begin{array}{c} \begin{array}{cc} TPR & =\frac{TP}{TP+FN},\\ TNR & =\frac{TN}{TN+FP}, \end{array} \end{array}$$ (26)

where TP, FP, TN and FN, stand for true positive, false positive, true negative, and false negative, respectively. It is important to remark the classification is done for expected returns in relation to historical returns, i.e., considering the asymmetric dependencies of historical returns as the benchmark structure.

Table 3 shows the TPR and TNR for each of the four models discussed so far. The classification is done by transforming the ETE real values represented in Fig 5 into binary values. Under this transformation if a ETE value is bigger than zero then it is set as one, otherwise, the ETE value is kept as zero. It is worth noting the TPR is very low, while TNR reaches values above 0.9 for all forecasting strategies. At this point, we can argue that neither model have enough power to preserve the TPR, while all of them maintain a high TNR. Then, since the point of view of asymmetric dependencies on large horizon forecasting models the selection of one or another model is irrelevant and we can select the one that meets the specific criterion needed for further analysis.

**Table 3:** Classification metrics for asymmetric dependencies. TPR and FNR for the binary ETE of estimated expected returns in relation to the binary ETE of historical returns.

Case	TPR	TNR
EGARCH(1,1)	0	0.9096
GJR-GARCH(1,1)	0.1667	0.9158
GARCH(1,1)	0.1	0.914
SBBS	0	0.9115

## 7 Multiperiod allocation

In this section, we present the results for the multiperiod allocation using the different estimations of the returns and its covariance matrices. In this order, seven experiments were performed considering the SBBS, GARCH(1,1), EGARCH(1,1) and the GJR-GARCH(1,1) models. These experiments are described in Table 4. Here, the third column indicate if one covariance matrix is estimated for all periods (Mono) or if one covariance matrix is estimated for each period (Multi).

**Table 4:** Experiments for the multiperiod allocation.

ID	Model	Covariance Matrix
E1	SBBS	Mono
E2	GARCH(1,1)	Mono
E3	GARCH(1,1)	Multi
E4	EGARCH(1,1)	Mono
E5	EGARCH(1,1)	Multi
E6	GJR-GARCH(1,1)	Mono
E7	GJR-GARCH(1,1)	Multi

In order to solve the multi-allocation model, we use the modeler YALMIP and the quadprog solver for MATLAB. The experiments were performed using MATLAB 2015 over a computer with 64GB of RAM and an Intel Core i9-9900K processor. The parameter *α* for the constraints (11) is fixed to 0.03 in the experiments since this is the best value with respect to the smoothness of the solutions. Furthermore, the weights *γ* for the variance in the objective function were considered in such a way that in the initial 22 periods are fixed to zero and in the last periods the weights increase linearly with the condition that must sum one. These values were considered in this way because it is expected that at the beginning the retirement fund can be exposed to risk in order to increase the wealth; but at the last periods the wealth of the people should be assured so it must face lower risk. Finally, the initial amount of money **x**(0) is generated considering one unit distributed uniformly over the assets. Finally, the algorithm takes 2 hours and 45 minutes, in average, to obtain the solutions of each experiment, which is relatively low considering that the inversion strategy is computed over 40 years.

**Fig 6:**
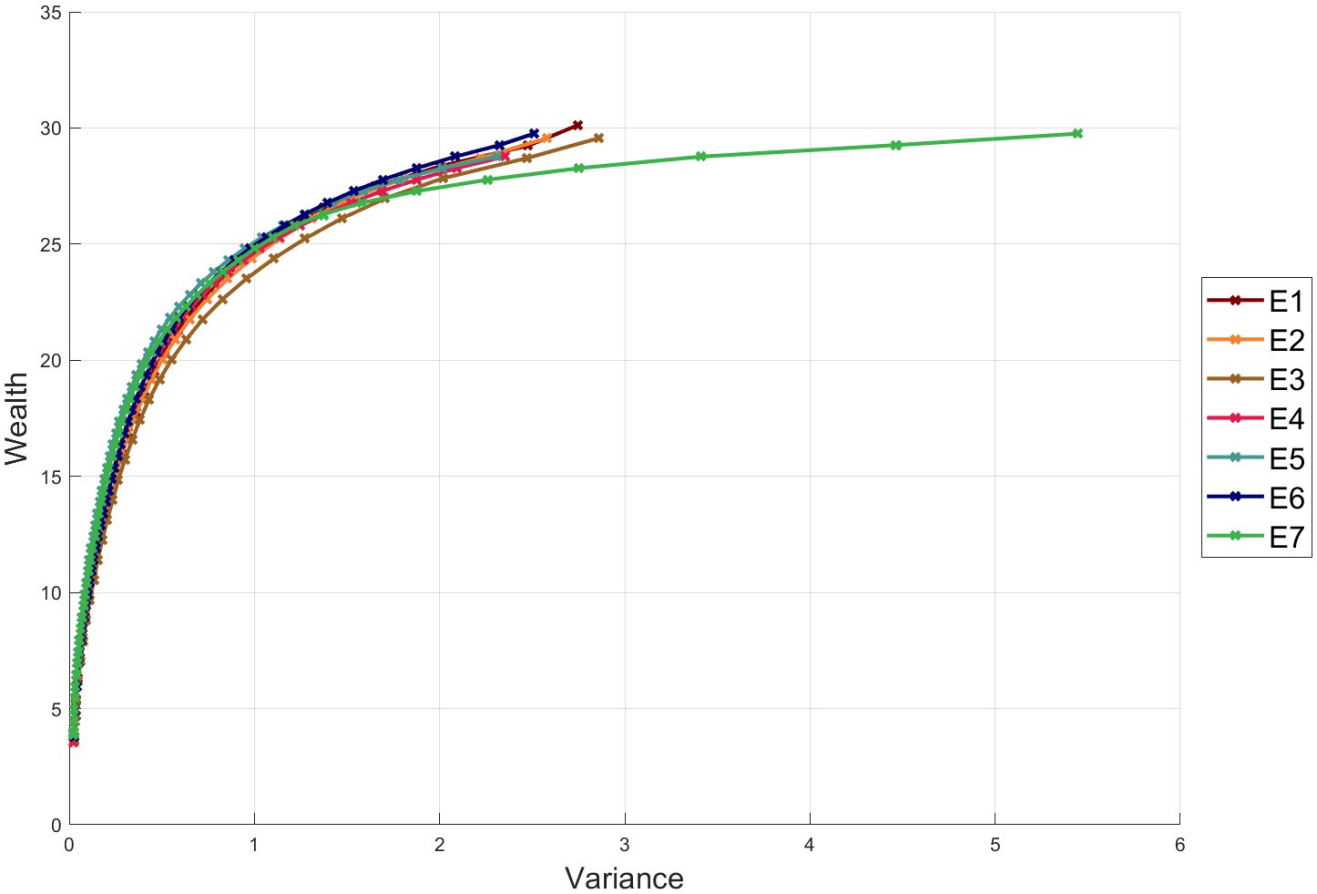
Pareto efficient allocations.

Fig 6 shows the efficient solutions for the experiments E1, E2, E3, E4, E5, E6 and E7, respectively. As can be seen, there is no significant difference among the solutions; the biggest difference can be observed in E7, which has a similar wealth; but a greater variance. The experiments show that for the SBBS, GARCH and EGARCH model there is not a significant change in the value of the variance in the respective experiments, although it is expected that the Multi experiments provide better estimations of the covariance matrices since they are estimated for each period in the investment horizon. In addition, the GJR-GARCH models E6 and E7 have the same wealth; but E7 has a slightly greater variance.

**Fig 7:**
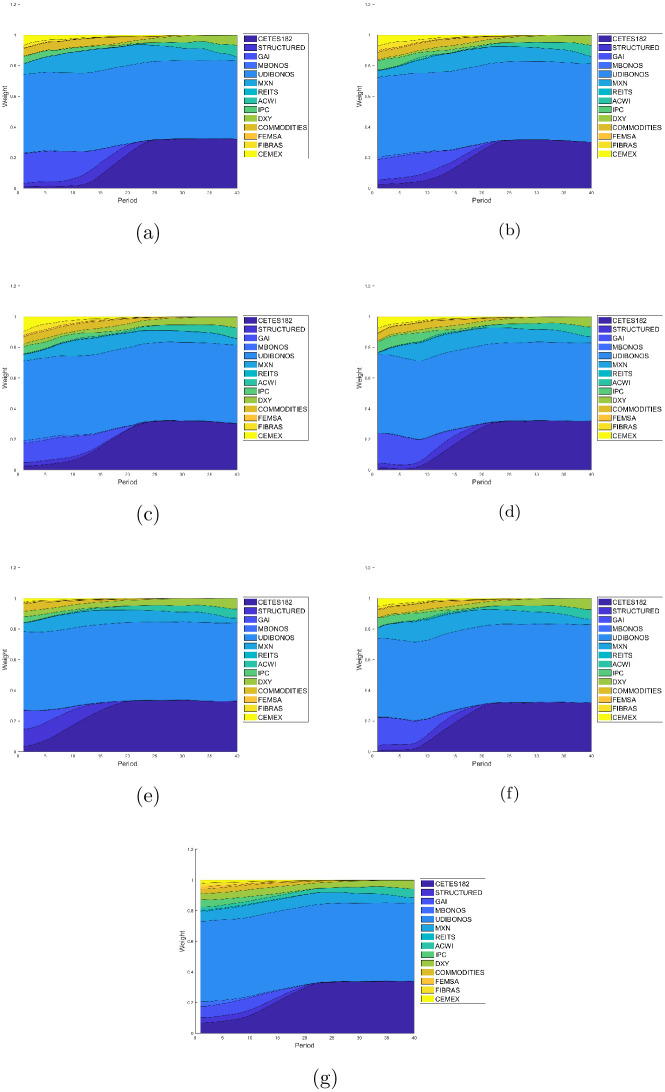
Allocation weights from the solution at the first quartile. (a) Case E1 with *E*{*W*(*T*)} = 7.1765 and *var*{*W*(*T*)} = 0.0570. (b) Case E2 with *E*{*W*(*T*)} = 7.0592 and *var*{*W*(*T*)} = 0.0549. (c) Case E3 with *E*{*W*(*T*)} = 7.0591 and *var*{*W*(*T*)} = 0.0607. (d) Case E4 with *E*{*W*(*T*)} = 7.4455 and *var*{*W*(*T*)} = 0.0568. (e) Case E5 with *E*{*W*(*T*)} = 8.9328 and *var*{*W*(*T*)} = 0.0665. (f) Case E6 with *E*{*W*(*T*)} = 7.9412 and *var*{*W*(*T*)} = 0.0646. (g) Case E7 with *E*{*W*(*T*)} = 8.9331 and *var*{*W*(*T*)} = 0.0722.

**Fig 8:**
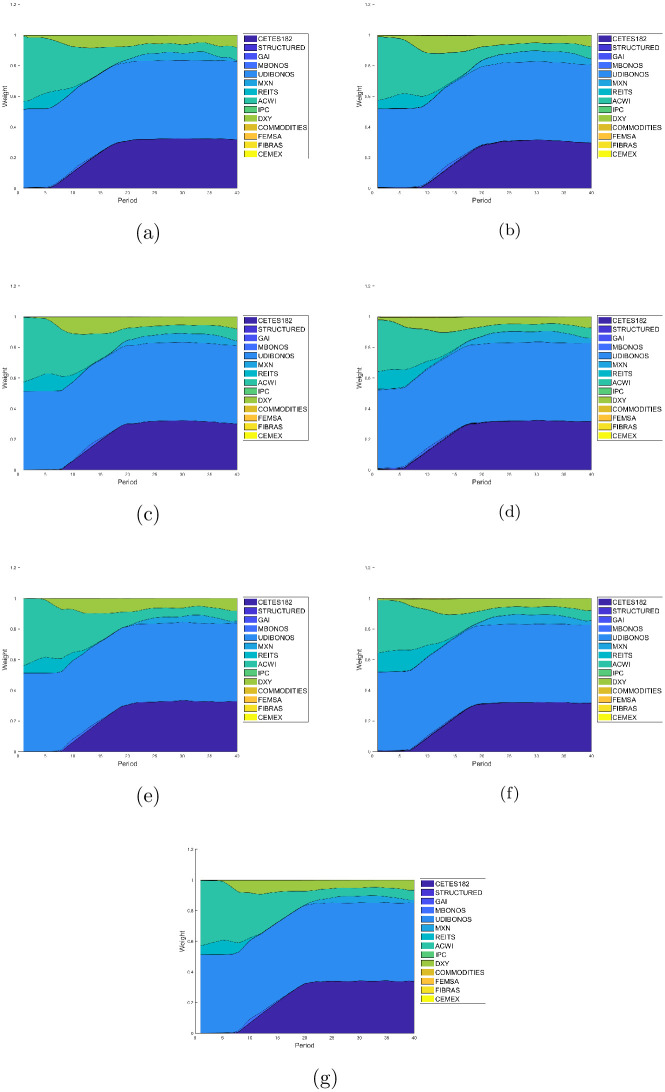
Allocation weights from the solution at the second quartile. (a) Case E1 with *E*{*W*(*T*)} = 15.1176 and *var*{*W*(*T*)} = 0.2502. (b) Case E2 with *E*{*W*(*T*)} = 14.8490 and *var*{*W*(*T*)} = 0.2436. (c) Case E3 with *E*{*W*(*T*)} = 14.8487 and *var*{*W*(*T*)} = 0.2652. (d) Case E4 with *E*{*W*(*T*)} = 14.3866 and *var*{*W*(*T*)} = 0.2111. (e) Case E5 with *E*{*W*(*T*)} = 15.8739 and *var*{*W*(*T*)} = 0.2179. (f) Case E6 with *E*{*W*(*T*)} = 15.3782 and *var*{*W*(*T*)} = 0.2436. (g) Case E7 with *E*{*W*(*T*)} = 15.8740 and *var*{*W*(*T*)} = 0.2312.

**Fig 9:**
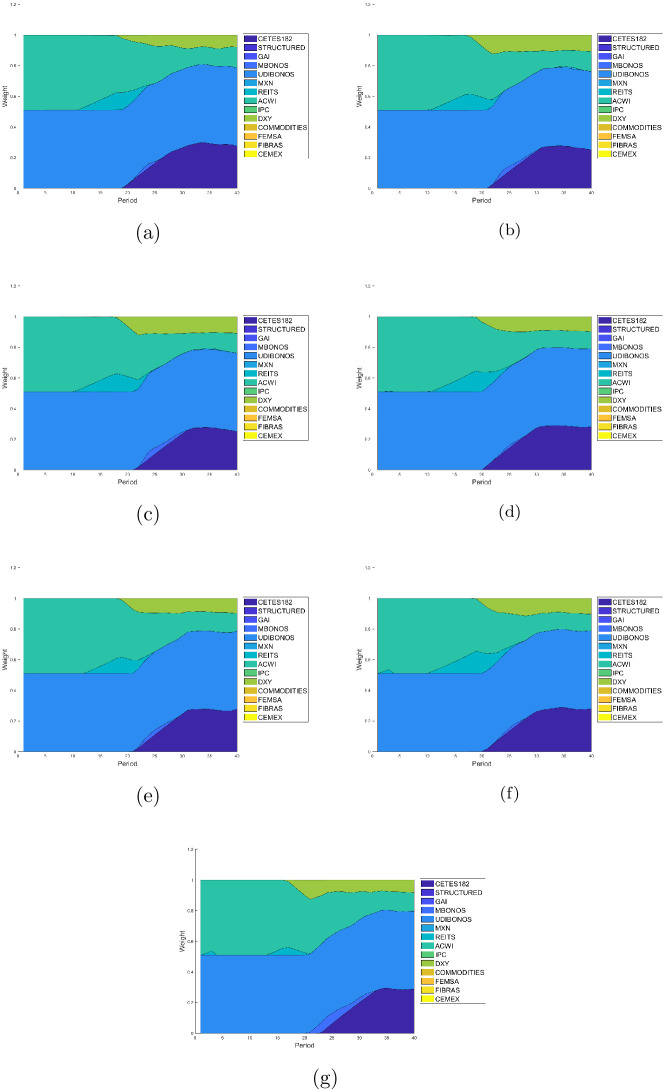
Allocation weights from the solution at the third quartile. (a) Case E1 with *E*{*W*(*T*)} = 22.1764 and *var*{*W*(*T*)} = 0.6683. (b) Case E2 with *E*{*W*(*T*)} = 21.7731 and *var*{*W*(*T*)} = 0.6512. (c) Case E3 with *E*{*W*(*T*)} = 21.7731 and *var*{*W*(*T*)} = 0.7222. (d) Case E4 with *E*{*W*(*T*)} = 21.8235 and *var*{*W*(*T*)} = 0.6088. (e) Case E5 with *E*{*W*(*T*)} = 22.3193 and *var*{*W*(*T*)} = 0.5934. (f) Case E6 with *E*{*W*(*T*)} = 22.3193 and *var*{*W*(*T*)} = 0.6408. (g) Case E7 with *E*{*W*(*T*)} = 22.8151 and *var*{*W*(*T*)} = 0.6901.

**Fig 10:**
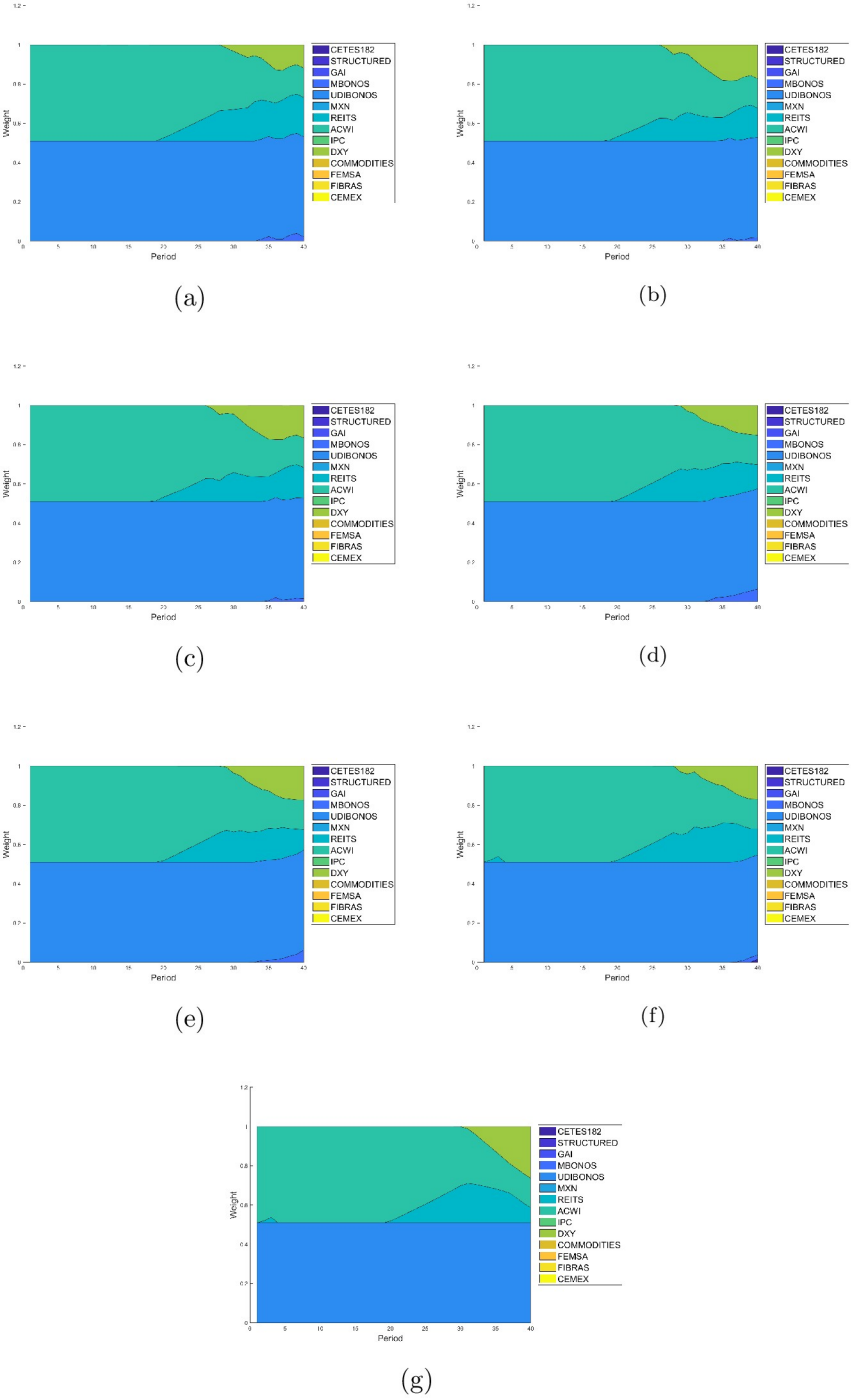
Allocation weights from the solution at the last quartile. (a) Case E1 with *E*{*W*(*T*)} = 29.2352 and *var*{*W*(*T*)} = 2.4781. (b) Case E2 with *E*{*W*(*T*)} = 28.6974 and *var*{*W*(*T*)} = 2.2212. (c) Case E3 with *E*{*W*(*T*)} = 28.6974 and *var*{*W*(*T*)} = 2.4701. (d) Case E4 with *E*{*W*(*T*)} = 28.2689 and *var*{*W*(*T*)} = 2.0927. (e) Case E5 with *E*{*W*(*T*)} = 28.2689 and *var*{*W*(*T*)} = 2.0181. (f) Case E6 with *E*{*W*(*T*)} = 29.2605 and *var*{*W*(*T*)} = 2.3225. (g) Case E7 with *E*{*W*(*T*)} = 29.2605 and *var*{*W*(*T*)} = 4.4609.

Figs 7–10 present the allocation weights for the solution of each quartile in terms of the wealth obtained for E1, E2, E3, E4, E5, E6 and E7, respectively. The final wealth and variance of the respective solution are shown in the title of each image. Observe that the solutions obtained from the returns estimated with the GJR-GARCH model reach an slightly greater wealth in comparison with the ones obtained with the other models to estimate the returns. Furthermore, the distributions of the investment are very similar among all the experiments, which means that the forecasts made with the different models are also similar. As expected, the solutions tend to make greedy decisions in the distribution with the maximum wealth, i.e., the algorithm tries to invest the most in the assets with the highest return at each period of the investment horizon.

The two lower quartiles in all experiments end up with over 80% of its assets coming from the debt market. Indeed, UDIBONOS and CETES182 replace COMMODITIES, IPC, GAI, and REITS in the long run. Actually, we can see that risky assets tend to be eliminated from the portfolio very early in the glide-path; as soon as 10-15 years. The third quartile is still conformed as the first two, in the end; but tends to include risky investment in the middle of its glide-path, starting around year 10 and reinvesting in it around 5–15 years. Finally, the fourth quartile restricts its share of bonds to 50% (UDIBONOS) and includes a greater part of risky assets. We see CETES182 being replaced by IPC, ACWI retaining a much greater participation in the final mix, and the FOREX market increasing its share via DXY.

In general, we see a simplification of the portfolio in the glide-path of the optimal investment for lower quartiles. Indeed, lower quartiles tend to be more diverse in the beginning, including small amounts of all assets; but with time, the capital is allocated mostly in five assets: UDIBONOS, CETES182, MXN, DXY, and ACWI. On the contrary, higher quartiles are less diverse at the start of the investment period and include diversification towards the end of the glide-path.

**Fig 11:**
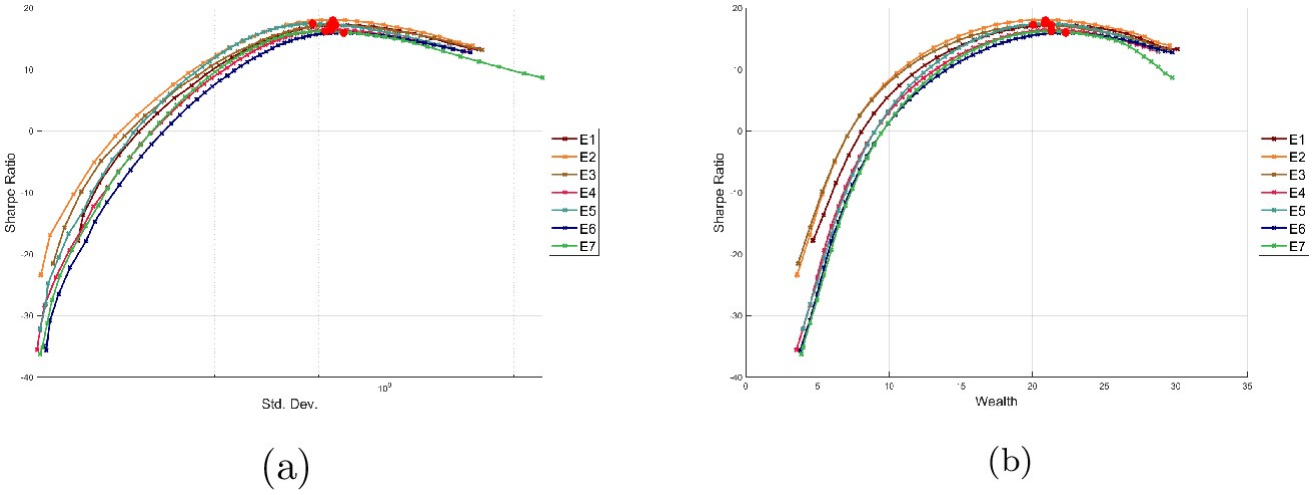
Sharpe ratio of the cases E1, E2, E3, E4, E5, E6 and E7. (a) Plot of Sharpe ratio and standard deviation. (b) Plot of Sharpe ratio and wealth.

In order to provide a strategy for the selection of an solution, we propose to consider the maximum Sharpe ratio [53], which represents the solution with the best trade-off between the total wealth and variance. In particular, the following equation is considered

$$\begin{array}{c} SR=\frac{R_{p}-R_{f}}{\sigma_{p}}, \end{array}$$

where *R*_*p*_ and *σ*_*p*_ are the return and the standard deviation of the portfolio for a given solution, and *R*_*f*_ represents the risk-free rate. In this work, the asset CETES182 is considered to compute *R*_*f*_. Fig 11 show the plots of the behavior of the Sharpe ratio over the fronts obtained in the experiments E1, E2, E3, E4, E5, E6 and E7. The red points in the figure represent the solutions with the maximum Sharpe ratio of each front.

**Fig 12:**
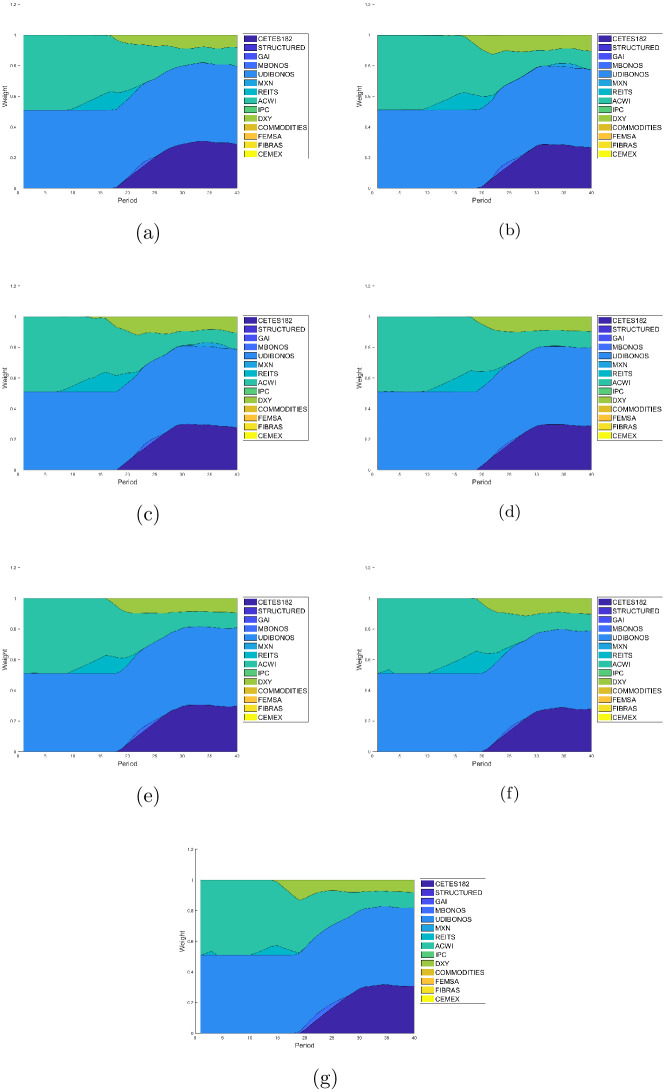
Allocation weights from the solution with the highest Sharpe ratio. (a) Case E1 with *E*{*W*(*T*)} = 21.2941 and *var*{*W*(*T*)} = 0.5862. (b) Case E2 with *E*{*W*(*T*)} = 20.9075 and *var*{*W*(*T*)} = 0.5725. (c) Case E3 with *E*{*W*(*T*)} = 20.0420 and *var*{*W*(*T*)} = 0.5541. (d) Case E4 with *E*{*W*(*T*)} = 21.3277 and *var*{*W*(*T*)} = 0.5619. (e) Case E5 with *E*{*W*(*T*)} = 20.8319 and *var*{*W*(*T*)} = 0.4588. (f) Case E6 with *E*{*W*(*T*)} = 22.3193 and *var*{*W*(*T*)} = 0.6408. (g) Case E7 with *E*{*W*(*T*)} = 21.3277 and *var*{*W*(*T*)} = 0.5285.

In addition, Fig 12 present the weights of the solution with the highest Sharpe ratio of each case, where the respective wealth and variance is shown.

We can see E2 reach the highest Sharpe ratio corresponding to the GARCH(1,1) model with fixed historical covariance matrix. Note the solutions show a balance between low and medium risk inversions. As expected in the early stages the portfolio allocate roughly half of their wealth on ACWI and REITS, whilst starting at the tenth year the glide-path gradually allocate mostly UDIBONOS and CETES182. This behaviour coincides with the glide-paths found on the literature and applied by practitioners, where is expected a high allocation of risk assets at early stages and a dominant allocation of bonds as the investment horizon reach its final stage.

## 8 Conclusions

The challenges posed to dynamically optimizing allocation in investment portfolios by the new regulations to the Mexican pension system put statistical and computational modeling to the test. Accounting for the many and diverse factors that affect the overall return of investment, while simultaneously computing the optimal glide-path calls for the use and synthesis of several complementary techniques. In this paper, we have shown that an adequate mixture of statistics, econometrics, and dynamic optimization provides a step forward in the solution to this problem. Specifically, we assumed a dynamic self-financing portfolio and minimized risk, as measured by weighted volatility, in each period. We evaluated the performance of this method using long-horizon bootstrap forecasts of each individual asset in the portfolio for three different volatility specifications as well as a stationary block bootstrap simulation as a baseline model for comparison purposes.

The dependence of the results we get on the particular GARCH specifications used in the simulations is shown to be minimal. Indeed, the SBBS, GARCH(1,1), EGARCH(1,1) and GJR-GARCH(1,1) experiments reveal very similar glide-paths so that the overall structure of the investment is maintained. This is a very desirable property of the model since it suggests we do not need to worry about model uncertainty when forecasting volatility. The optimal investment was also robust to the model employed for the correlation matrix of the returns.

Overall, most of the solutions capture the property of allocation of risky assets at early stages and a dominance of bonds at the final steps. Nevertheless, the glide-paths found at the highest Sharpe ratio show the most parsimonious solution in the sense of the best trade-off between wealth and variance. In particular the GARCH(1,1) experiment with fixed historical covariance matrix represents the best solution in terms of risk aversion.

The relatively low computational cost granted by the reduction of the multistage allocation problem to a convex quadratic programming problem with linear constraints makes this method applicable. The inclusion of the risk weighting constants *γ*(*k*), *k* ≥ 1 provides flexibility and allows for the inclusion of risk ratings, risk management strategies, or regulatory constraints.

The fact that a simpler GARCH(1,1) model reaches the higher Sharpe ratio and the TPR and TNR of the asymmetric dependencies measured with transfer entropy did not present remarkable variations between the different forecasting models motivates us to hypothesize the next. In long-range multi-step optimization, the most important matter is that the dependencies of the assets are maintained. Thus, we could relax some stylized assumptions about financial returns in order to reduce the complexity of the forecasting models and instead focus on improving the optimization strategy. This is certainly something that should be studied in future work systematically. Also, interesting future research may include even more realistic settings possibly including contributions and withdrawals as well as tax commissions and their practical implications in the replacement rate on the Mexican pensioners.

## Supporting information

S1 File Data. Returns of assets used in this study as described in the data section.
(CSV)
